# Impact on hospital ranking of basing readmission measures on a composite endpoint of death or readmission versus readmissions alone

**DOI:** 10.1186/s12913-017-2266-4

**Published:** 2017-05-05

**Authors:** Laurent G. Glance, Yue Li, Andrew W. Dick

**Affiliations:** 1 Department of Anesthesiology, University of Rochester School of Medicine, Rochester, USA; 2 RAND Health, RAND, Boston, USA; 3 Department of Public Health Sciences, University of Rochester School of Medicine, Rochester, USA

## Abstract

**Background:**

Readmission penalties are central to the Centers for Medicare and Medicaid Services (CMS) efforts to improve patient outcomes and reduce health care spending. However, many clinicians believe that readmission metrics may unfairly penalize low-mortality hospitals because mortality and readmission are competing risks. The objective of this study is to compare hospital ranking based on a composite outcome of death or readmission versus readmission alone.

**Methods:**

We performed a retrospective observational study of 344,565 admissions for acute myocardial infarction (AMI), congestive heart failure (CHF), or pneumoniae (PNEU) using population-based data from the New York State Inpatient Database (NY SID) between 2011 and 2013. Hierarchical logistic regression modeling was used to estimate separate risk-adjustment models for the (1) composite outcome (in-hospital death or readmission within 7-days), and (2) 7-day readmission. Hospital rankings based on the composite measure and the readmission measure were compared using the intraclass correlation coefficient and kappa analysis.

**Results:**

Using data from all AMI, CHF, and PNEU admissions, there was substantial agreement between hospital adjusted odds ratio (AOR) based on the composite outcome versus the readmission outcome (intraclass correlation coefficient [ICC] 0.67; 95% CI: 0.56, 0.75). For patients admitted with AMI, there was moderate agreement (ICC 0.53; 95% CI: 0.41, 0.62); for CHF, substantial agreement (ICC 0.72; 95% CI: 0.66, 0.78); and for PNEU, substantial agreement (ICC 0.71; 95% CI: 0.61, 0.78). There was moderate agreement when the composite and readmission metrics were used to classify hospitals as high, average, and low-performance hospitals (*κ* = 0.54, SE = 0.050). For patients admitted with AMI, there was slight agreement (*κ* = 0.14, SE = 0.037) between the two metrics.

**Conclusions:**

Hospital performance on readmissions is significantly different from hospital performance on a composite metric based on readmissions and mortality. CMS and policy makers should consider re-assessing the use of readmission metrics for measuring hospital performance.

**Electronic supplementary material:**

The online version of this article (doi:10.1186/s12913-017-2266-4) contains supplementary material, which is available to authorized users.

## Background

Nearly 20% of Medicare patients are readmitted after hospital discharge with an estimated annual cost of $26 billion [1, 2]. Hospital readmission rates are highly variable across hospitals, even after adjusting for patient risk [1, 3, 4]. Many readmissions are preventable [5, 6] and high hospital readmission rates are thought to reflect poor quality of care. Patient safety events [7] and postoperative complications [8, 9] are often the result of poor-quality care, and lead to unplanned readmissions. Improving the coordination of care, better discharge planning, and early physician follow-up can lower readmission rates [10, 11]. The Hospital Readmission Reduction Program (HRRP) was established by the Affordable Care Act in 2012 to create financial incentives for hospitals to lower hospital readmissions. Under the HRRP, hospitals with risk-adjusted readmission rates that are greater than the rate of readmissions predicted for an average hospital with the same patient case mix can lose up to 3% of their CMS payments [12]. Two-thirds of U.S. hospitals were subject to payment penalties of $428 million in 2014–2015 under this program [2, 13]. Although originally focused on medical conditions – heart failure, myocardial infarctions, and pneumoniae – the HRRP has been expanded to include surgical procedures – total hip arthroplasty, total knee arthroplasty, and coronary artery bypass graft surgery (CABG).

Making hospitals accountable for poor quality care has face validity, and is at the center of CMS efforts to stop paying simply for the volume of care (i.e. fee-for-service) and instead pay for the outcomes or quality of care. But the use of readmission metrics to assess quality of care has been criticized [14] because it ignores the fact that hospital mortality rates and readmission rates are poorly correlated, and that low-mortality hospitals frequently have high readmission rates [15–17]. Inpatient mortality and readmission are competing risks since patients who die during their initial hospital stay or after discharge cannot be readmitted [18]. Low-mortality hospitals may discharge sicker patients than higher-mortality hospitals, and these sicker patients may be more likely to require re-hospitalization [19]. Under the current CMS HRRP, all hospitals with excess readmissions are penalized, irrespective of their inpatient mortality rates.

Our goal in this exploratory study is to determine whether giving hospitals credit for fewer deaths would significantly impact hospital rankings based on readmissions. We first constructed a novel hospital readmission metric based on the composite outcome of in-hospital death or readmission. Using this composite outcome, hospitals with fewer deaths and more readmissions will not, by construction, necessarily appear to deliver lower-quality care compared to hospitals with fewer readmissions and more deaths. We assumed that this composite outcome more accurately reflects quality of care than a performance metric based on readmission outcomes alone. We then compared hospital performance on this composite outcome versus the conventional metric based on readmissions alone. The findings of this study may help CMS and other policy makers, as well as measure developers, explore the impact of revising readmission metrics to remove the “death bonus” from hospital readmission metrics.

## Methods

### Data source

We conducted this study using data (2011–2013) from the Health Cost and Utilization Project State Inpatient Database (HCUP SID) for New York State (NYS). The NYS SID includes data on all inpatient discharges from non-Federal short-term acute-care hospitals in NYS [20]. The NYS SID contains information on patient demographic characteristics, admission source, type of admission (urgent, emergency, and elective), ICD-9-CM diagnostic and procedure codes, Agency for Healthcare Research and Quality comorbidity measures [21], payer source, in-hospital mortality (30-day mortality is not available in the HCUP data), and hospital identifiers. The NYS SID also includes a synthetic person-level identifier and a timing variable that can be used to track hospital readmission [22]. This study was approved by the Research Subjects Review Board at the University of Rochester and a waiver of consent was granted (RSRB00061697).

### Patient population

Our analysis included patients admitted with a primary diagnosis of acute myocardial infarction (AMI), congestive heart failure (CHF), or pneumonia (PNEU). Patients admitted with one of these primary diagnoses >7 days after the index hospitalization were counted as a new index hospitalization [15]. For each index admission, only discharge records for which patients survived to discharge contributed to the hospital readmission metric, whereas all patient discharge records contributed to the composite outcome. By construction, the same patient could have several index admissions included in the analysis. We identified 380,015 adult patient records (age ≥ 18) with a primary admission diagnosis of AMI, CHF, or PNEU. Patients discharged in December of 2013 were excluded from our analysis (11,388) because their 7-day readmission status was not available in our data. We excluded patients who were discharged against medical advice (4,812), transferred to another acute care hospital (16,532), or with hospital length-of-stay greater than 365 days (147). We also excluded 2,571 patients with missing information on race, payer status, emergency status, or discharge status. Our final patient sample included 344,565 records from 204 hospitals.

### Model specification

For our baseline analysis, we first specified a readmission model using hierarchical logistic regression for all-cause 7-day readmissions. We used the 7-day time window for readmissions in order for the mean rate of readmission and the mean rate of in-hospital mortality to be approximately equal – as they are for AMI, CHF, and PNEU when using the 30-day time window for both readmissions and mortality [15]. For example, the in-hospital mortality rate for AMI in our sample was 5.81% and the 7-day readmission rate was 6.75%. Using Medicare data, Krumholz and colleagues showed that the mean risk standardized 30-day mortality rate for AMI was 16.6%, and the mean risk standardized 30-day readmission rate for AMI 19.9% [15]. To assess the potential impact of basing the CMS readmission metric on a composite outcome of death and readmission, it was important to insure that the relative contribution of death and readmission to the composite metric were similar.

Hospitals were specified as a random intercept. Only patients who survived to hospital discharge were included. We adjusted for patient demographics (age, gender, race and ethnicity), urgent or emergency admission, payer status, comorbidities, transfer-in status, and primary admission diagnosis (AMI, CHF or PNEU). We adjusted for comorbidities using the AHRQ Elixhauser algorithm instead of the CMS algorithm because our analysis was based on inpatient discharge data, and we did not have access to outpatient claims data. The AHRQ Elixhauser comorbidity algorithm is widely used in risk adjustment models based on administrative data [21, 23–25]. We used fractional polynomials to obtain the optimal specification for age [26]. We did not impute missing data because the number of patients with missing data was less than 1% of our sample. The empirical-Bayes estimate of the hospital random effect was exponentiated to estimate the hospital adjusted odds ratio (AOR). The hospital AOR estimates the probability that a patient admitted to a specific hospital will be readmitted within 7-days of discharge, controlling for patient risk. Hospitals were categorized as high-performance outliers if their AOR is significantly less than 1 (*P* < 0.05), and as low-performance outliers if their AOR is significantly greater than 1 (*P* < 0.05).

We then estimated a death or readmission model with a composite outcome model of 7-day readmission or in-hospital death using the same methodology used for the readmission model described above. This second model did not exclude patients who died before hospital discharge.

We then estimated separate models for AMI, CHF, and PNEU as described above.

### Statistical analysis

We performed several hospital-level analyses to quantify the level of agreement between the readmission measures versus the composite measure. We examined the level of agreement for the hospital AORs using intraclass correlation coefficient. We used weighted Kappa analysis to assess the level of agreement for categorical measures (high-performance, average-performance, and low-performance outliers) of hospital quality [27]. The extent of agreement is k <0.0, poor agreement; 0.0-0.2, slight agreement; 0.21-0.40, fair agreement; 0.41-0.6, moderate agreement; 0.61-0.8, substantial agreement; 0.81-1.0, almost perfect agreement [28]. We used the same scale to grade the level of agreement using the ICC. We performed these analyses for all patients together (AMI, CHF, and PNEU), and then separately for each cohort of AMI, CHF, PNEU patients.

Database management and all statistical analyses were performed using STATA SE/MP Version 14.1 (STATA Corp., College Station, TX). Hierarchical modeling was performed using xtmelogit in STATA. We also performed a sensitivity analysis in which we used melogit (STATA) and estimated the empirical mean Bayes estimates of the random effects (instead of the empirical mode Bayes estimates using xtmelogit). Model performance was evaluated using measures of discrimination (C statistic) and calibration (Brier statistic).

## Results

### Study population

The study sample consisted of 344,565 admissions to 204 hospitals (Table 1). The rate of in-hospital mortality or 7-day readmissions (composite outcome) was 10.93%, and the rate of 7-day readmissions was 6.75%. For patients admitted with AMI, the study sample consisted of 75,036 patients with a 11.81% rate of in-hospital mortality or 7-day readmissions, and 6.36% rate of 7-day readmissions; for CHF, 144,338 patients with a 11.31% rate of in-hospital mortality or 7-day readmissions, and 7.71% rate of 7-day readmissions; for pneumonia, 125,191 patients with a 9.97% rate of in-hospital mortality or 7-day readmissions, and 5.86% rate of 7-day readmissions (Table 1).

**Table 1:** Distribution of Hospital Adjusted Odds Ratio for Composite Outcome and Readmission

Principle Discharge Diagnosis	No. of Patients	No. of Hospitals	Rate (unadj)	Median Odds Ratio	AOR, Interquartile Range	AOR, Range	Estimated Between Hospital Variance
AMI, CHF, or PNEU							
Composite Outcome	344,565	204	10.93	1.37	0.88-1.10	0.48-1.66	0.0316
Readmission	329,120	204	6.75	1.17	0.93-1.07	0.69-1.43	0.0079
Acute Myocardial Infarction							
Composite Outcome	75,036	188	11.80	1.22	0.93-1.08	0.59-1.44	0.0128
Readmission	70,677	188	6.36	1.15	0.96-1.03	0.70-1.26	0.0068
Congestive Heart Failure							
Composite Outcome	144,338	200	11.31	1.35	0.88-1.11	0.51-1.51	0.0297
Readmission	138,715	200	7.71	1.16	0.94-1.05	0.73-1.37	0.0075
Pneumoniae							
Composite Outcome	125,191	204	9.97	1.22	0.91-1.11	0.57-1.48	0.0126
Readmission	119,728	204	5.86	1.18	0.94-1.07	0.74-1.44	0.0088

*AOR*, adjusted odds ratio, *AMI* acute myocardial infarction, *CHF* congestive heart failure, *PNEU* pneumonia

### Variation in hospital performance

The percentage of the variability in patient outcome caused by differences in hospital performance was quantified using the intraclass correlation coefficient (ICC) [29]. In the study sample, hospitals accounted for 3.2% of the variability in the composite outcome, and 0.79% of the variability in 7-day readmissions (Table 1 and Additional file 1: Appendix Table 1). For AMI, hospitals accounted for 1.3% of the variability in the composite outcome, and for 0.68% of the variability in readmissions; for CHF, 3.0% of the variability in the composite outcome, and 0.75% of the variability for readmissions; and, for pneumonia, 1.26% of the variability in the composite outcome, and 0.88% of the variability in readmissions.

The hospital median odds ratio (MOR) [29] is the median of the distribution of the ratio of 2 randomly selected hospital AORs, and reflects the increased risk of the outcome of interest attributable to hospital selection: the larger the MOR, the greater the variation in hospital performance. In the overall sample, the MOR for the composite outcome was 1.37, and the MOR for readmissions was 1.17. For AMI, the MOR for the composite outcome was 1.22, and the MOR for readmissions was 1.15; for CHF, 1.35 for the composite outcome, and 1.16 for readmissions; and for pneumonia, 1.22 for the composite outcome, and 1.18 for readmissions.

Model discrimination, as assessed using the C statistic, was not very good (C statistic ranging between 0.60 and 0.71), but was consistent with the performance of readmission prediction models (Table 2) [30]. Model calibration, as measured using the Brier score was acceptable (Brier score ranged between 0.056 and 0.12 (Table 2).

**Table 2:** Model Performance

	C statistic	Brier Score	LR test for RE effect
Principle Discharge Diagnosis			
AMI, CHF, or PNEU			
Composite Outcome	0.67	0.093	<0.0001
Readmission	0.62	0.062	<0.0001
Acute Myocardial Infarction			
Composite Outcome	0.71	0.098	<0.0001
Readmission	0.64	0.059	<0.0001
Congestive Heart Failure			
Composite Outcome	0.64	0.097	<0.0001
Readmission	0.60	0.071	<0.0001
Pneumoniae			
Composite Outcome	0.69	0.086	<0.0001
Readmission	0.63	0.055	<0.0001

**Table 3:** Hospital Performance based on the Composite Outcome versus Readmission Performance Metric

	Readmission or death				
	High-performance	Average-performance	Low-performance		
	AMI, CHF, or PNEU			Hospital AOR, median (range)	Kappa
Readmission, No. (%)					0.54
High-performance	12 (80.0)	3 (20.0)	0 (0)	0.81 (0.69-0.90)	
Average-performance	23 (14.0)	130 (79.3)	11 (6.7)	0.99 (0.81-1.17)	
Low-performance	0 (0)	5 (20.8)	19 (79.2)	1.24 (1.13-1.43)	
Hospital AOR, median (range)	0.79 (0.48-0.92)	0.99 (0.63-1.46)	1.33 (1.16-1.66)		
	Acute Myocardial Infarction				0.13
Readmission, No. (%)					
High-performance	2 (100)	0 (0)	0 (0)	0.76 (0.70-0.81)	
Average-performance	16 (8.7)	164 (88.7)	5 (2.7)	1.00 (0.81-1.26)	
Low-performance	0 (0)	1 (100)	0 (0)	1.23 (NA)	
Hospital AOR, median (range)	0.80 (0.59-0.88)	1.02 (0.78-1.35)	1.41 (1.23-1.44)		
	Congestive Heart Failure				0.53
Readmission, No. (%)					
High-performance	8 (88.9)	2 (11.1)	0 (0)	0.80 (0.73-0.88)	
Average-performance	10 (5.6)	158 (88.3)	11 (6.15)	1.00 (0.83-1.23)	
Low-performance	0 (0)	3 (27.3)	8 (72.7)	1.29 (1.13-1.37)	
Hospital AOR, median (range)	0.77 (0.51-0.86)	0.98 (0.70-1.39)	1.33 (1.16-1.51)		
	Pneumoniae				0.45
Readmission, No. (%)					
High-performance	4 (66.7)	2 (33.3)	0 (0)	0.77 (0.74-0.81)	
Average-performance	7 (3.7)	168 (88.9)	14 (7.4)	0.99 (0.83-1.31)	
Low-performance	0 (0)	1 (12.5)	7 (87.5)	1.27 (1.21-1.44)	
Hospital AOR, median (range)	0.76 (0.57-0.81)	0.99 (0.76-1.44)	1.34 (1.16-1.48)		

*NA* not applicable, *No* number, *AOR* adjusted odds ratio, *AMI* acute myocardial infarction, *CHF* congestive heart failure, *PNEU* pneumonia

kappa – weighted kappa analysis

### Hospital ranking as a function of readmission versus composite metric

**Fig. 1:**
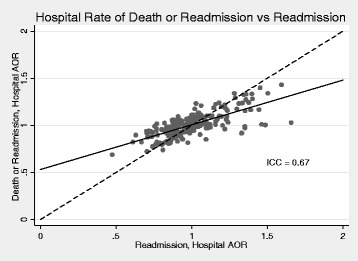
Agreement between proposed readmission metric based on composite outcome of 7-day readmission and in-hospital mortality versus standard readmission metric based on 7-day readmissions. The hospital AOR represents the likelihood that patients admitted with AMI, CHF or pneumonia at a specific hospital are likely to experience the outcome of interest compared to the “average” hospital, after adjusting for patient case mix. The identity (*dashed*) line represents perfect agreement, and the *solid* line is a regression line fitted to the data

**Fig. 2:**
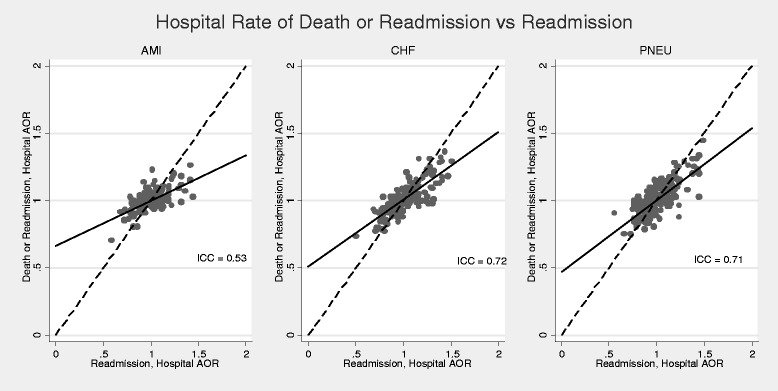
Agreement between proposed readmission metric based on composite outcome of 7-day readmission and in-hospital mortality versus standard readmission metric based on 7-day readmissions. The hospital AOR represents the likelihood that patients admitted with either AMI, CHF or pneumonia at a specific hospital are likely to experience the outcome of interest compared to the “average” hospital, after adjusting for patient case mix. The identity (*dashed*) line represents perfect agreement, and the *solid* line is a regression line fitted to the data

We quantified the amount of agreement between hospital rankings as a function of the readmission outcome versus the composite outcome using the intraclass correlation coefficient. An ICC of 1.0 indicates perfect agreement. In the baseline analysis, there was substantial agreement between hospital AOR based on the composite outcome versus the readmission outcome (ICC 0.67; 95% CI: 0.56 – 0.75) (Fig. 1 and Additional file 1: Appendix Figure 1). For patients admitted with AMI, there was moderate agreement (ICC 0.53; 95% CI: 0.0.41 – 0.62); for CHF, substantial agreement (ICC 0.72; 95% CI: 0.66 – 0.78); and for PNEU, substantial agreement (ICC 0.71; 95% CI: 0.61 – 0.78) (Fig. 2).

We then quantified the level of agreement between hospital performance (high-performance, average-performance, and low-performance) based on the composite metric versus the readmission metric hospital classification using the weighted kappa statistic. In the baseline analysis, there was moderate agreement between the two metrics (*κ* = 0.54, SE = 0.050) (Table 3). For patients admitted with AMI, there was slight agreement (*κ* = 0.13, SE = 0.037); for CHF, there was moderate agreement (*κ* = 0.53, SE = 0.048); and for PNEU, there was moderate agreement (*κ* =0.45, SE = 0.046).

## Discussion

In this population-based exploratory study of patients admitted with acute myocardial infarction, congestive heart failure, or pneumonia, we find evidence that hospitals’ performance on readmissions is significantly different from their performance on a composite metric based on readmissions and mortality. About one-fifth of hospitals classified as average-performance hospitals using the readmission metric were re-classified as high or low-performance hospitals using the composite metric. Overall, we found moderate agreement when hospital quality was assessed using mortality and readmissions versus readmissions alone. In the case of patients admitted with acute myocardial infarction, there was slight agreement. Our findings should raise concern that the CMS Hospital Readmission Reduction Program (HRRP) may not accurately classify high and low-performance hospitals for the purpose of value-based purchasing.

Hospital readmissions alone may not be an accurate measure of quality of care. Some low-mortality hospitals may discharge a greater number of sicker patients, who are more likely to be readmitted, compared to high-mortality hospitals [14]. These hospitals may be unfairly penalized by the HRRP. On the other hand, some high-mortality hospitals with high rates of failure-to-rescue may discharge fewer sick patients, and these patients may be less likely to be readmitted. Using the current readmission metric, these high-mortality hospitals may “appear” to be delivering high-quality care because they have fewer readmissions. Others have proposed combining mortality and readmission into a single quality measure [14, 19]. Our findings provide empiric support for the claim that hospital readmission metrics, as currently specified, may not accurately reflect hospital quality.

Our findings may have important policy implications. Quality measurement is at the center of efforts to improve the quality and lower the cost of health care. But, quality measurement is an imperfect science [31]. The quality of quality measurement is critically dependent on many factors such as data quality [32] and risk adjustment [33]. The impact of data quality on quality assessment has been widely studied by comparing hospital assessment using lower-quality administrative data versus higher-quality clinical data [34]. The choice of which risk factors to include in risk adjustment has a profound effect on which hospitals are identified as high or low-performance [35]. Similarly, the choice of statistical methodology – standard versus hierarchical modeling – also has a major impact on hospital ranking [36]. In some ways, the choice of outcomes to use for quality assessment seems more straightforward and intuitive, and therefore less controversial. The use of hospital readmissions as a quality indicator was driven by the frequency and cost of hospital readmissions. However, unlike other quality indicators, the use of hospital readmissions to measure hospital quality has been repeatedly questioned [14, 18, 19, 37]. In particular, many clinicians are uncomfortable with the fact that hospitals with lower mortality rates and high readmission rates are subject to financial penalties under CMS’s HRRP. Krumholz and colleagues have shown that since hospital readmission rates and mortality rates are poorly correlated [15], they measure different quality domains. Irrespective of whether hospital performance should be assessed using a single composite based on mortality and readmissions, or 2 separate metrics – one for readmissions and one for mortality, using readmission alone as the basis for payment penalties is unfair because it does not take into account rates of hospital mortality. The success of value-based purchasing will be determined, at least in part, by the face validity of the quality indicators used to measure the quality of care. Our findings provide empiric support for the widely-held assumption that readmission metrics may be flawed because they fail to account for patient mortality. Our findings, along with the work by Krumholz, suggest that CMS should not base the HRRP on readmission outcomes alone.

This study has several important limitations. First, our choice of outcome was limited by the use of the NYS State Inpatient Database. We would have preferred to use a composite of (1) in-hospital mortality or mortality within 30-days of discharge, and (2) readmission within 30-days of discharge. Furthermore, because the NYS SID provides information only on in-hospital mortality, and not 30-day mortality, we would have under-estimated the impact of including death in the composite outcome had we used in-hospital mortality in conjunction with 30-day readmission. However, by selecting a time window of 7 days for readmissions, instead of 30-days, we were able to preserve the relative magnitude of readmissions and mortality in our analysis [15]. Thus, our findings – based on in-hospital mortality and 7-day readmissions - should be qualitatively similar to those obtained using 30-day mortality and 30-day readmissions. In addition, because our study does not consider mortality after hospital discharge, and since patients who die following hospital discharge cannot be readmitted, it is likely that our results underestimate the discordance in hospital performance based on death or readmission versus readmission alone. Nonetheless, our findings need to be replicated using 30-day mortality and 30-day readmission data.

Second, our composite outcome gives equal weight to mortality and readmission. Using our composite outcome, Hospital A – with a mortality of 3% and readmission rate of 5% - will be considered equivalent to Hospital B – with a mortality of 5% and readmission rate of 3%. These hospitals are clearly not equivalent. However, the current CMS approach using readmissions alone, would label the higher-mortality hospital in this example as higher quality.

Third, our readmission model does not account for the fact that readmission is conditional on survival. Patients discharged alive may have unobserved risk that is different than that of patients who die in the hospital, leading to sample selection bias [38]. In practice, approaches to mitigate sample selection bias are not commonly used in the literature for readmission models, nor are they used by CMS. Furthermore, our model for the composite outcome does account for inpatient mortality by including mortality as an outcome. So all patients admitted to the hospital are included in the analysis whether they die in the hospital or are discharged alive and at risk for readmission. Therefore our composite model eliminates the possibility of selection bias due to inpatient mortality.

Fourth, our study is based on administrative data. Although the limitations of administrative data are well known, CMS uses readmission metrics based on administrative data in the HRRP. Model discrimination, as measured using the C statistic, although not very good, is consistent with values obtained by others [30]. In part, the relatively poor performance of readmission models may be caused by the absence of many important patient social factors that may play an important role in hospital readmissions [30]. However, since the main purpose of our study is to examine the impact of modifying existing CMS readmission measures on quality assessment, and CMS readmission measures are based on administrative data, issues with data quality are not a significant limitation of this pragmatic study. Finally, our study was limited to New York State and may not be generalizable to other parts of the U.S. However, there is no reason to assume, a priori, that hospitals in other states would not exhibit qualitatively similar differences in apparent quality if performance assessment is based on readmissions versus death and readmissions.

CMS publicly reports readmission and mortality metrics separately to measure two quality domains. Supporters of the current reporting policy argue that readmission metrics are not intended to measure quality as stand-alone measures, which is why readmission metrics are reported alongside with mortality metrics and do not need to incorporate mortality. But, prior work has shown that not accounting for the correlation between mortality and readmissions leads to selection bias [38]. Our findings provide further evidence that not accounting for the correlation between readmissions and mortality may lead to a biased measure of quality.

## Conclusions

In summary, whether hospital performance is assessed using readmissions alone versus readmissions and mortality may have a significant impact on hospital ranking. We believe that physician efforts to improve the quality of health care will be strongest, and most effective, when clinicians believe that performance assessment reflects true clinical quality. Our empiric findings, coupled with a strong clinical rationale for not basing hospital quality assessment on readmissions alone, should prompt CMS and policy makers to re-assess the use of existing readmission metrics that do not account for patient mortality. Future studies should also include post-discharge mortality to examine the full impact of expanding readmission metrics to include deaths on hospital ranking.

## Additional file

Additional file 1:
**Figure S1.** Agreement between proposed readmission metric based on composite outcome of 7-day readmission and in-hospital mortality versus standard readmission metric based on 7-day readmissions. **Table S1.** Hospital Performance based on the Composite Outcome versus Readmission Performance Metrics (sensitivity analysis based on empirical mean Bayes estimates). (DOCX 18 kb)

## Abbreviations

AMI: Acute myocardial infarction
AOR: Adjusted odds ratio
CHF: Congestive heart failure
CMS: Centers for Medicare and Medicaid Services
HCUP: Health Cost and Utilization Project
HRRP: Hospital Readmission Reduction Program
ICC: Intraclass correlation coefficient
k: Kappa statistic
MOR: Median odds ratio
NY SID: New York State Inpatient Database
NYS: New York State
PNEU: Pneumoniae
SE: Standard error
TX: Texas
