# Supplementary material for: Impact on hospital ranking of basing readmission measures on a composite endpoint of death or readmission versus readmissions alone
Source: BMC Health Serv Res. 2017 May 5;17:327. doi: 10.1186/s12913-017-2266-4 (PMC5420148; doi:10.1186/s12913-017-2266-4)
Supplement: Additional file 1: — Figure S1. Agreement between proposed readmission metric based on composite outcome of 7-day readmission and in-hospital mortality versus standard readmission metric based on 7-day readmissions. Table S1. Hospital Performance based on the Composite Outcome versus Readmission Performance Metrics (sensitivity analysis based on empirical mean Bayes estimates). (DOCX 18 kb) [file 12913_2017_2266_MOESM1_ESM.docx]

**APPENDIX**

**Appendix Figure 1.** Agreement between proposed readmission metric based on composite outcome of 7-day readmission and in-hospital mortality versus standard readmission metric based on 7-day readmissions.

**Appendix Table 1.** Hospital Performance based on the Composite Outcome versus Readmission Performance Metrics (sensitivity analysis based on empirical mean Bayes estimates).

| **Appendix Table 1.** Hospital Performance based on the Composite Outcome versus Readmission Performance Metrics (sensitivity analysis based on empirical mean Bayes estimates). | | | | | |
| --- | --- | --- | --- | --- | --- |
|  | **Readmission or death** | | |  | |
|  | **High-performance** | **Average-performance** | **Low-performance** |  |  |
|  | **AMI, CHF, or PNEU** | | | Hospital AOR,  median (range) | Kappa |
| **Readmission, No. (%)** |  |  |  |  | 0.54 |
| High-performance | 12 (75.0) | 4 (25.0) | 0 (0) | 0.81 (0.69-0.90) |  |
| Average-performance | 23 (14.0) | 130 (79.3) | 11 (6.7) | 0.99 (0.81-1.17) |  |
| Low-performance | 0 (0) | 4 (17.4) | 19 (82.6) | 1.24 (1.13-1.43) |  |
| Hospital AOR,  median (range) | 0.79 (0.48-0.92) | 0.99 (0.63-1.46) | 1.34 (1.16-1.65) |  |  |

NA – not applicable; No – number; AOR – adjusted odds ratio; AMI – acute myocardial infarction; CHF – congestive heart failure; PNEU – pneumonia

Kappa – weighted kappa analysis

**Appendix Figure** Agreement between proposed readmission metric based on composite outcome of 7-day readmission and in-hospital mortality versus standard readmission metric based on 7-day readmissions. (Sensitivity analysis using empirical mean Bayes estimates of the hospital random effects)**.**

The hospital AOR represents the likelihood that patients admitted with AMI, CHF or pneumonia at a specific hospital are likely to experience outcome of interest at the “average” hospital, after adjusting for patient case mix. The identity (dashed) line represents perfect agreement, and the solid line is a regression line fitted to the data.
